# Multi-Omics Reveals Different Strategies in the Immune and Metabolic Systems of High-Yielding Strains of Laying Hens

**DOI:** 10.3389/fgene.2022.858232

**Published:** 2022-04-01

**Authors:** Muhammad Arsalan Iqbal, Henry Reyer, Michael Oster, Frieder Hadlich, Nares Trakooljul, Alvaro Perdomo-Sabogal, Sonja Schmucker, Volker Stefanski, Christoph Roth, Amélia Camarinha Silva, Korinna Huber, Vera Sommerfeld, Markus Rodehutscord, Klaus Wimmers, Siriluck Ponsuksili

**Affiliations:** ^1^ Research Institute for Farm Animal Biology, Institute of Genome Biology, Dummerstorf, Germany; ^2^ University of Hohenheim, Institute of Animal Science, Stuttgart, Germany; ^3^ University Rostock, Faculty of Agricultural and Environmental Sciences, Rostock, Germany

**Keywords:** data integration, mixOmics, microbiota, miRNA, mRNA, immune cells, metabolites, laying hen

## Abstract

Lohmann Brown (LB) and Lohmann Selected Leghorn (LSL) are two commercially important laying hen strains due to their high egg production and excellent commercial suitability. The present study integrated multiple data sets along the genotype-phenotype map to better understand how the genetic background of the two strains influences their molecular pathways. In total, 71 individuals were analyzed (LB, *n* = 36; LSL, *n* = 35). Data sets include gut miRNA and mRNA transcriptome data, microbiota composition, immune cells, inositol phosphate metabolites, minerals, and hormones from different organs of the two hen strains. All complex data sets were pre-processed, normalized, and compatible with the mixOmics platform. The most discriminant features between two laying strains included 20 miRNAs, 20 mRNAs, 16 immune cells, 10 microbes, 11 phenotypic traits, and 16 metabolites. The expression of specific miRNAs and the abundance of immune cell types were related to the enrichment of immune pathways in the LSL strain. In contrast, more microbial taxa specific to the LB strain were identified, and the abundance of certain microbes strongly correlated with host gut transcripts enriched in immunological and metabolic pathways. Our findings indicate that both strains employ distinct inherent strategies to acquire and maintain their immune and metabolic systems under high-performance conditions. In addition, the study provides a new perspective on a view of the functional biodiversity that emerges during strain selection and contributes to the understanding of the role of host–gut interaction, including immune phenotype, microbiota, gut transcriptome, and metabolome.

## Introduction

The two commercially important laying hen strains, Lohmann Brown (LB) and Lohmann Selected Leghorn (LSL), are selected for high egg production (Singh et al., 2009; Habig et al., 2012; Reyer et al., 2021). Although LB and LSL are nearly identical in egg production performance, these strains differ considerably in other phenotypic traits including body weight, gene expression, immunological traits, bone metabolism, and gastrointestinal phytate degradation (Kaufmann et al., 2011; Silversides et al., 2012; Sommerfeld et al., 2020; Ponsuksili et al., 2021).

Previously, comparative transcriptomics from brain tissue revealed that while transcripts’ upregulation contributed to immune system processes in LSL, the downregulation was involved in phosphorus (P) metabolism and signaling pathways (Habig et al., 2012). Following dietary interventions with reduced P and calcium (Ca) intake, the intestinal transcriptional profile of the two strains showed strain-specific alterations, for example, in the cell proliferation rate and extracellular matrix formation, which might also be due to different mineral and vitamin D requirements of LB and LSL (Reyer et al., 2021). Similarly, the miRNAs expression profiles from the jejunum of LB and LSL hens fed with different amounts of Ca and P indicate that miRNA targets contributed to metabolic pathways in LB; miRNA targets were involved in Ca signaling pathways and mitochondrial dysfunction in LSL (Iqbal et al., 2021). In addition, the deep-sequenced miRNAs of the jejunum of LB and LSL at different production periods demonstrated that miRNAs play a pivotal role in regulating gene expression and impacting intestinal homeostasis differently in both strains (Ponsuksili et al., 2021).

Several studies have suggested that the host’s genetic background is a factor that might influence gut microbiota composition (Org et al., 2015; Schokker et al., 2015; Han et al., 2016; Kers et al., 2018). The host intestinal epithelia and gut microbiota ecosystem are complex and consist of diverse molecular activities, including immune and metabolic functions (Simon et al., 2016; Broom and Kogut, 2018; Borda-Molina et al., 2021). Recent studies demonstrated a shift in the microbiota during the laying hens’ lifespan or change in metabolite profiles between the LB and LSL strains (Gonzalez-Uarquin et al., 2021; Joat et al., 2021). Concerns about the environment, nutrient supply, and farm profit contribute to the increasing attention to the Ca and mineral P supplements in animal feed (Delezie et al., 2015). Numerous studies indicated that adding additional P and Ca to the feed of broiler chickens significantly reduced endogenous Inositol hexakisphosphate (InsP_6_) degradation (Tamim et al., 2004; Shastak et al., 2014; Zeller et al., 2015). Compared with broilers, laying hens need less P in their diet, but they require substantially more Ca because of eggshell formation (Ahmadi and Rodehutscord, 2012). Consequently, the processes of InsP_6_ degradation, myo-inositol (MI) release, and P/Ca utilization might be more distinct in laying hens than in broilers (Sommerfeld et al., 2020; Sommerfeld et al., 2020). Sommerfeld et al. (2020) described the effects of dietary Ca and P on intestinal phytate degradation and mineral utilization during the laying phase in LB and LSL and also elaborated that measured traits depend on the hen strain. It was concluded that to meet their respective mineral demands, LB and LSL use different mechanisms, including transcellular transport in LB and more effective phytate degradation in LSL. In addition, results indicated that the variation in Ca and P concentration in feed affects the body weight of the LB strain, while no effect was observed in the LSL strain.

Since poultry production intensifies and antibiotics are under pressure to be used less frequently, maintaining and improving poultry health by promoting animal-intrinsic mechanisms become more relevant (Swaggerty et al., 2019). Here, factors such as nutrition and genetics play a critical role in modulating immunity in commercial poultry production (Koenen et al., 2002; Kidd, 2004; Kjærup et al., 2017; Nie et al., 2018; Hofmann et al., 2021). Interestingly, laying hens fed higher nonphytate-P levels were found to have higher interferon (IFN) levels in the blood, suggesting an improved immune system (Nie et al., 2018).

Using advancements in high-throughput approaches and the availability of multiple omics data generated from similar and different experiments, data integration provides the material for a more comprehensive biological interpretation at multiple levels, which can support the unfolding of the complex biological processes scientifically and holistically (Subramanian et al., 2020). An earlier study identified a highly correlated multi-omics signature including host mRNA, miRNA, and microbial data for identifying the molecular drivers for P utilization (PU) in Japanese quail (Ponsuksili et al., 2020) using the multi-block discriminant analysis with DIABLO (Data Integration Analysis for Biomarker discovery using a Latent cOmponents) embedded in the R package “mixOmics” (Rohart et al., 2017; Singh et al., 2019).

In our preliminary work, individual datasets covering physiological data, hormones, metabolites, immune traits, host transcriptome, or microbiome were retrieved from the same animals of the LB and LSL strains in separate work packages of the DFG (Deutsche Forschungsgemeinschaft) Research Unit “P-Fowl” in the context of divergent dietary Ca and P supply (Sommerfeld et al., 2020; Hofmann et al., 2021; Iqbal et al., 2021; Reyer et al., 2021). Individual analysis of each dataset for the two laying hen strains in the various studies revealed specific differences at each level and lacked an integrated multi-omics view of these specific physiological changes. The integration of multi-omics provides a new perspective on the functional biodiversity that arises during strain selection and contributes to our understanding of the role of host–gut interactions. In the present study, we integrate these datasets in a holistic biological analysis to characterize the functional biodiversity of the two strains that contribute to attaining comparable performance *via* different molecular mechanisms. Finally, strain-specific bio-signatures are being uncovered to deepen our understanding of the relationships between underlying immunology, genetics, metabolism, developmental processes, and gut microbial community composition.

## Materials and Methods

### Experimental Design and Sample Selection

We used previously published datasets (Sommerfeld et al., 2020; Hofmann et al., 2021; Iqbal et al., 2021; Reyer et al., 2021). In brief, hatchlings of brown (LB) and white (LSL) layer strains, representing two distinct genetic backgrounds, were obtained from a breeding company (Lohmann Tierzucht GmbH, Cuxhaven, Germany). LB and LSL were selected for their egg production performance while being monitored for bone quality, egg quality, and behavior (Preisinger, 2018). During rearing, the standard management conditions for the pullet phase of the experiment station of the University of Hohenheim were applied (Sommerfeld et al., 2020). All hens received identical corn-soybean meal-based diets meeting or exceeding the supply recommendations of the breeding company for the starter, grower, pre-laying, and layer phases. From week 27 to 31, forty individuals per strain received experimental diets with reduced Ca, P, or a combination thereof, as previously described by Sommerfeld et al. (2020). In week 31, the blood and intestinal contents were sampled. Trunk blood or vein blood was collected depending on the targets of analysis (immune features, metabolites, P, Ca, MI, or hormones). Tissue samples for transcriptome analysis were collected from jejunum mucosa, while digesta and mucosa from the duodenum were collected for the microbiota investigations. Since the study design was balanced between LB and LSL in terms of diet and the effects of strain clearly outweighed the dietary alterations [as reported earlier (Sommerfeld et al., 2020; Iqbal et al., 2021; Reyer et al., 2021)], the dietary groups were combined in the present study for the downstream analysis. Specifically, residuals were calculated for each parameter after adjusting for diet and father effects and used for further downstream analysis.

### Data Pre-Processing

The miRNA and mRNA expression profiles were gathered from the jejunum. The read count matrices of miRNA and mRNA from the same birds were obtained from our previous studies under accession numbers (E-MTAB-9136) and (E-MTAB-9109), respectively (Iqbal et al., 2021; Reyer et al., 2021). The samples with lower counts, that is, outliers deviating from the mean by more than two SD, were excluded. Finally, the total number of samples analyzed in this study was *n* = 71, whereas *n* = 36 for LB and *n* = 35 for LSL. Microbiota data were represented as amplicon sequence variants (ASVs) that were deduced from 16S rRNA sequencing from the duodenal mucosa and the duodenal digesta of LB and LSL. Initially, ASVs were assigned to taxa at the genus level and were filtered so that only taxa with more than one observation in at least half of the samples were considered. Subsequently, miRNA and mRNA read count matrices and microbiota ASVs count matrices were transformed by variance-stabilizing transformation (VST) using the DESeq2 R package (Love et al., 2014).

Previously collected data from the same birds include immunological traits such as the counts of many types of T cells, B cells, heterophils, thrombocytes, and monocytes from blood, the spleen, and pharyngeal tonsils. Data on metabolites are from blood and digesta, including InsP3-6 isomers, P, Ca, MI, and hormones, and other phenotypic traits include Ca and P intake, Ca and P utilization, Ca and P excretion, feed intake, and body weight (Sommerfeld et al., 2020; Hofmann et al., 2021).

After pre-processing by transformation or normalization of each data type, data were adjusted for systemic effects using JMP Genomics (SAS Institute, Cary, NC, United States) mixed analyses of variance. Diet was used as a fixed effect and hen father as a random effect. The residuals after adjustment for diet and father were further used for the integration analysis.

### Data Integration

To integrate the data, we used the R package mixOmics (version.6.10.9) (Rohart et al., 2017). All preprocessed input data matrices were subjected to mixOmics for further filtering, removing predictors with zero or near-zero variance. We integrated six data blocks: mRNA (13,455 features), miRNA (185 features), immune system (54 features), metabolome (29 features), microbiome (111 features), and phenotypic features (11 features).

Data integration, classification, feature selection, and visualization were carried out by Data Integration Analysis and Biomarker discovery using Latent variable approaches for Omics studies (DIABLO) (Singh et al., 2019). The DIABLO multi-omic approach instantly predicts significant biomarkers, including mRNAs, metabolites, proteins, and miRNA. DIABLO is the first multivariate integrative classification method to identify correlated or co-expressed features from heterogeneous data sets. We used the N-integration supervised Sparse Partial Least Square Discriminant Analysis (SPLS-DA) approach for feature selection (Lê Cao et al., 2011; Gley et al., 2021). The *block. splsda*() function was used to identify signatures composed of highly correlated variables across the multiple matrix sets, enabling us to detect a confident relationship between the data sets (Singh et al., 2019).

To evaluate the number of parameters and global performance, to select the appropriate metric distance, and to determine the number of components for our block. splsda analysis, we used DIABLO’s pref () function. The parameters set for the pref () function were Mfold validation (*n* = 5) and cross-validation (nrepeat = 10). For all supervised N-integration models, the tuning function was crucial for selecting the optimal components and features. We ran the tuning function tune. block.splsda () to predict the optimal number of features that were finally used for our block. splsda analysis. The best performance was obtained with the optimal component selection based on the balanced error rate (BER). The parameters used for the tune. block.splsda () function were M-fold validation (*n* = 5) and cross-validation (nrepeat = 10). In addition, the parameter distance metric for splsda to estimate the classification error rate (dist = max dist) and two misclassification measures, the total error rate and balanced error rate (BER) were used.

Subsequently, visualization of the block. splsda results was accomplished by various plotting functions in a mixomics environment. The discriminant analysis results were visualized by the *PlotArrow*() and *plotindiv*() functions. The *PlotArrow*() function plotted an arrow plot, which indicates the components’ scores from multiple datasets. It generates arrows between the scores associated with two or more groups, in our case, the two groups LB and LSL. In contrast, the *plotindiv*() function generates the individual plot, which depicts different data blocks, where each block sample shows differentiation between two or more groups. The components of the block. splsda results were visualized by the *plotLoadings*() function. The *plotLoadings*() function plotted loading plots that represent the loading weights of each feature selected on each dataset block. At the same time, the size bar indicates the significance of the selected feature between two or more groups. In addition, the bar’s color is associated with the group in which the selected features are most prevalent. A Circos correlation plot was generated by the *circosplot*() function; the plot demonstrates the significant signature from multiple datasets and their correlation coefficient. LB could be distinguished from LSL laying hens *via* two components of biomarkers from the heterogeneous data sets.

### Correlation Analysis of Bio-Signature Features and Gut mRNA Transcripts

The miRNAs selected using the above approaches (DIABLO) were used for the prediction of target mRNAs and the study of negative correlation. Therefore, variance-stabilized counts were used. A heatmap of bio-signature miRNAs selected from mixomics was generated using gplots (version 3.1.1) R package function *heatmap.2*() (Warnes et al., 2016). The scatter plot of differentially expressed mRNAs was visualized by *plot*() within the R programming environment (https://www.R-project.org/).

The miRNAs and their potential mRNA targets obtained from the recent chicken genome assembly (GRC6a) were predicted using RNAhybrid version 2.1.2 by setting the parameter binding energy with a cut-off of 25 k, the helix constraint in a range from 2 to 7, and one hit per target (Rehmsmeier et al., 2004). MiRNAs and their downstream mRNA targets were selected based on their minimum free energy and *p*-value, as previously described (Iqbal et al., 2021). The Pearson correlation was calculated between bio-signature miRNAs and differentially expressed (DE) mRNAs at FDR ≤ 5%. In addition, mRNAs inversely correlated with miRNAs were included for further downstream analysis. Moreover, strain-specific correlation analyses were performed between jejunal mRNA profiles and bio-signature revealed for data sets of immune cells, microbes, blood/digesta parameters (metabolites, P, Ca, MI, and hormones), and phenotypic traits. Pearson correlation was calculated between the complete set of mRNAs with datasets mentioned above within LB and LSL. The correlation was considered significant at FDR ≤ 5%.

### Gene Ontology and KEGG Pathway Enrichment Analysis

Functional annotation enrichment analysis was performed on the identified miRNAs and their inversely correlated DE mRNA targets. The mRNAs negatively correlated with miRNAs were subjected to ClueGO (version.2.5.1) and Cluepedia (version 1.5.7) plug-ins within Cytoscape (3.6.1.) for gene ontology (immune system processes) and Kyoto Encyclopedia of Genes and Genomes (KEGG) pathway enrichment analysis (Bindea et al., 2009; Saito et al., 2012; Bindea et al., 2013). Likewise, pairs of inversely correlated miRNAs/mRNAs were subject to DAVID (version 6.8) for gene ontology (biological processes) enrichment analysis (Huang et al., 2007). GOplot (version 1.0.2) within the R programming environment generates GO circular and Circos plots for gene ontology enrichment analysis (Walter et al., 2015). ClueGO was employed to plot a functionally annotated KEGG pathways network.

Moreover, gene ontology and KEGG pathway enrichment analyses were performed considering the complete set of jejunal mRNAs correlated with bio-signature of immune cells, microbes, blood/digesta parameters, and phenotypic traits at the cutoff criteria of FDR ≤ 0.05 and (r ≥ ± 0.5) within LB and LSL. For gene ontology and KEGG pathway enrichment analyses, these were further subjected to DAVID (version 6.8) and ClueGO, respectively. ClueGO revealed KEGG pathway enrichment networks, while DAVID results were imported to the ggplot2 R package to create dot plots for gene ontology enrichment analysis (Wickham et al., 2016). Parameters used for ClueGO and DAVID were right-sided hypergeometric tests to calculate the *p*-value, Benjamin–Hochberg for multiple testing correction, and *Gallus gallus* as a reference genome. The KEGG pathways, biological processes, and immune system processes with *p* ≤ 0.05 were considered significant.

## Results

**FIGURE 1:**
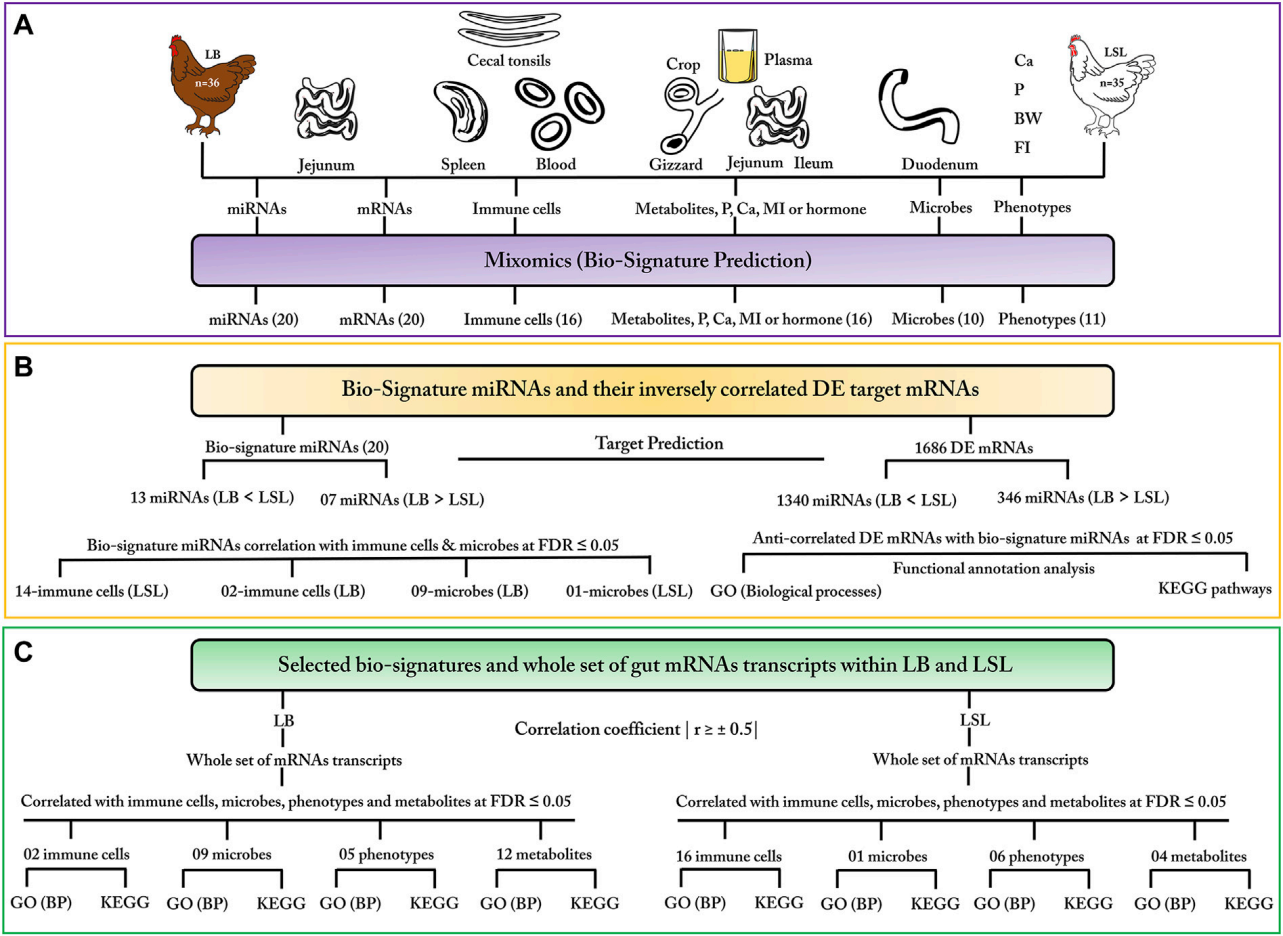
Framework for multi-omics data integration and downstream analysis. **(A)** Data types: miRNAs, mRNAs, immune cells, microbes, blood/digesta parameters, and phenotypic traits were measured from different tissues, blood, and plasma of the LB and LSL laying hen strains. Discriminant analysis in mixOmics was performed using the Sparse Partial Least Square Discriminant Analysis (SPLS-DA) supervised method for data classification and feature selection. The number of selected features in each dataset is given in brackets. **(B)** Pearson correlations between selected miRNAs and bio-signature of immune cells and microbes were calculated for LB and LSL at FDR ≤5%. Moreover, miRNA–mRNA negatively correlated pairs, derived from the twenty selected miRNAs and differentially expressed (DE) mRNAs in the jejunum, were subjected to functional annotation analysis. **(C)** Strain-specific bio-signatures were applied to correlation analysis and functional enrichment. Within LB and LSL, Pearson correlation was calculated between the whole set of gut mRNAs and bio-signatures of immune cells, microbes, phenotypic traits, and blood/digesta parameters at an FDR ≤5% and (r ≥ ± 0.5). Finally, mRNAs correlated with the different datasets were subjected to functional enrichment analysis.

Our study combines miRNA expression, mRNA expression, immune cell profiles, microbial composition, blood/digesta data (metabolites, P, Ca, MI, and hormones), and phenotypic traits from different tissues, blood, and plasma of LB and LSL chicken strains. The initial datasets included 80 birds. After pre-processing and filtration, data from 71 animals (36 from LB and 35 from LSL) were considered for further downstream analysis. We developed a framework for integrating these data sets into one analysis using the mixOmics platform and predicting significant bio-signatures from the heterogeneous dataset (Figure 1A). To examine the data variation between LB and LSL, we performed a discriminatory analysis using the Sparse Partial Least Square Discriminant Analysis (SPLS-DA) supervised approach available in the mixOmics R package. A mixOmics revealed the selection of the most discriminant features between the two laying hen strains, including 20 miRNAs, 20 mRNAs, 16 immune parameters, 10 microbes, 11 phenotypic traits, and 16 blood/digesta parameters (Figure 1A). In order to characterize the molecular and metabolic routes linked to these selected biomarkers, the individual features were further used for correlation with selected miRNAs and mRNAs pairs (Figure 1B) and with the whole set of intestinal transcripts (Figure 1C).

**FIGURE 2:**
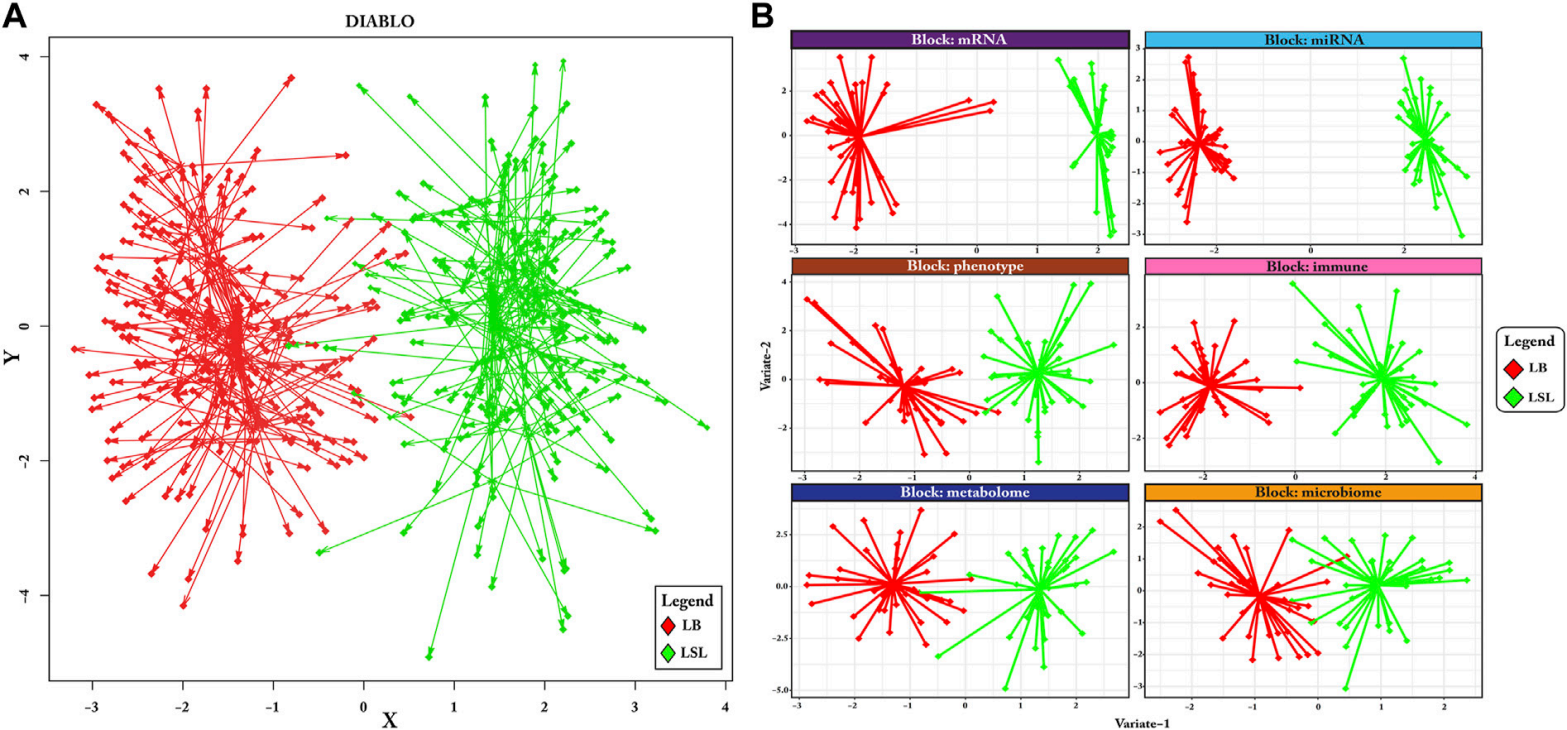
Discriminant analysis between the two strains of laying hens, LB and LSL. **(A)** The arrow plot indicates the discrimination of LB and LSL hens as depicted as consensus components based on the integration of all datasets. The start of the arrow represents the components’ score in the first dimension, while the diamond symbol on the arrow’s tip gives the components’ scores related to the second dimension for each individual. Short arrows indicate substantial agreement between the matching datasets, whereas long arrows depict disagreement between the matching datasets. The red and green colors represent the LB and LSL, respectively. **(B)** The individual plots depict six data blocks: miRNA, mRNA, phenotype, immune, metabolome, and microbiota, where each block sample showed differentiation between LB and LSL. The red is for LB and green is for LSL.

Considering the different datasets available for the laying hens, a considerable degree of separation between LB and LSL samples was achieved (Figure 2A). Discriminant analyses performed for the individual data blocks comprising miRNA, mRNA, immune traits, microbiome, blood/digesta parameters, and phenotypic traits showed a clear separation of groups (Figure 2B). Compared to other blocks, miRNA, mRNA, and immune parameters revealed the highest degree of separation between LB and LSL.

### Integration and Identification of Biomarkers Specifying the Lohmann Brown and Lohmann Selected Leghorn Strains

**FIGURE 3:**
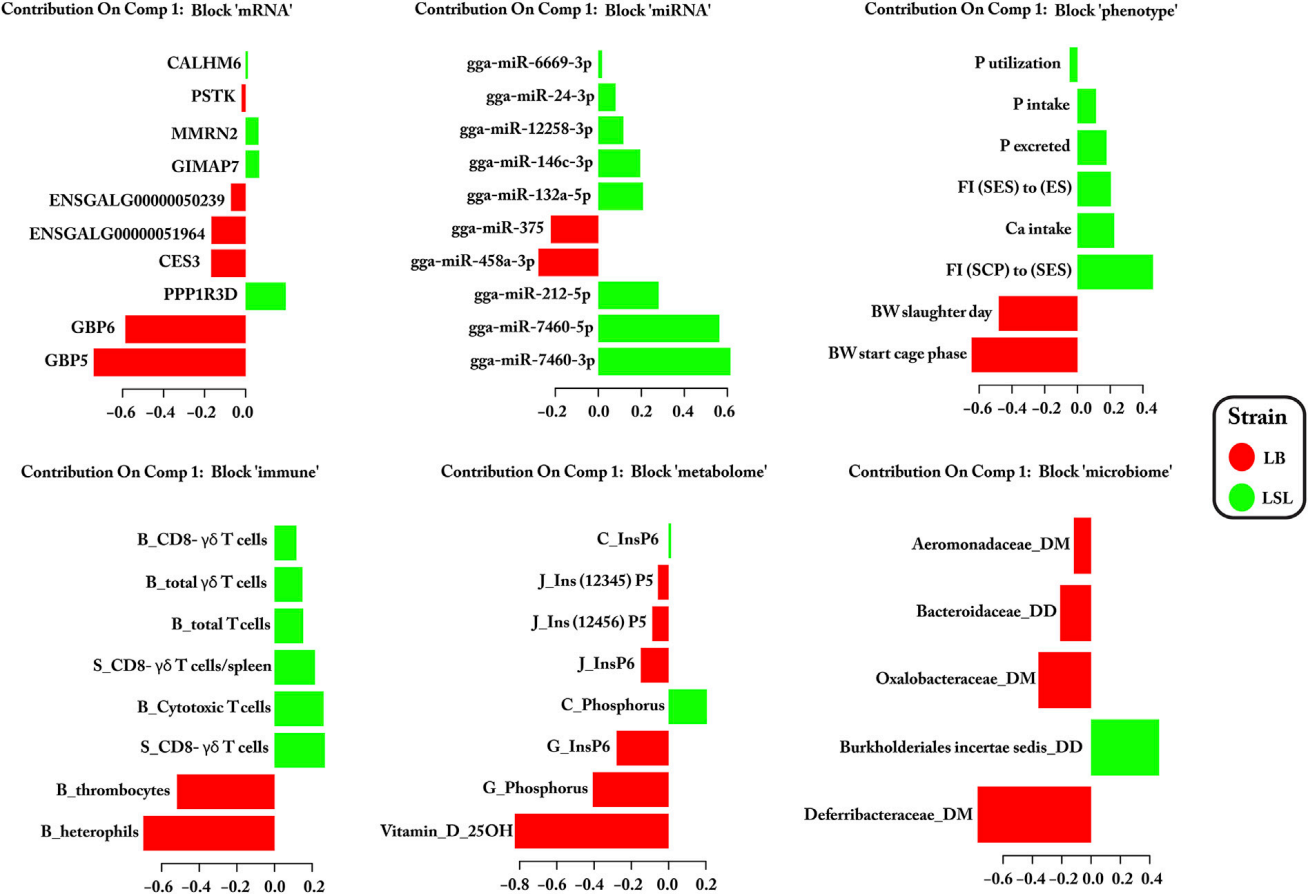
Significant biomarker loading weights for blocks of miRNAs, mRNAs, phenotypic traits, immune cells, metabolites, and microbes discriminating between LB and LSL over component 1. Barplot names correspond to the feature selected on each block: mRNA, miRNA, phenotype, immune traits [B (blood) or S (spleen) means the original organ from which these immune cells originate], metabolome [C (crop) or J (jejunum) or G (gizzard) means the original organ from which these metabolites originate], and microbiota (DD or DM means duodenum digesta and mucosa). The length of the bar represented the significance (loading weights) of the selected features, while the color is related to the strain in which the specific feature is most abundant.

**FIGURE 4:**
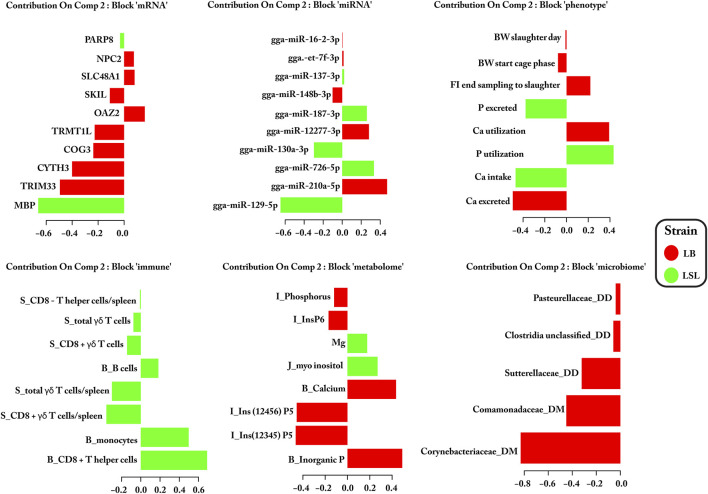
Significant biomarker loading weights for blocks of miRNAs, mRNAs, phenotypic traits, immune cells, metabolites, and microbes discriminating between LB and LSL over component 2. Barplot names correspond to the feature selected on each block: mRNA, miRNA, phenotype, immune traits [B (blood) or S (spleen) means the original organ from which these immune cells originate], metabolome [I (ileum) or J (jejunum) or B (blood) means the original organ from which these metabolites originate], and microbiota (DD and DM means duodenum digesta and mucosa). The length of the bar represented the significance (loading weights) of the selected features, while the color is related to the strain in which the specific feature is most abundant.

**FIGURE 5:**
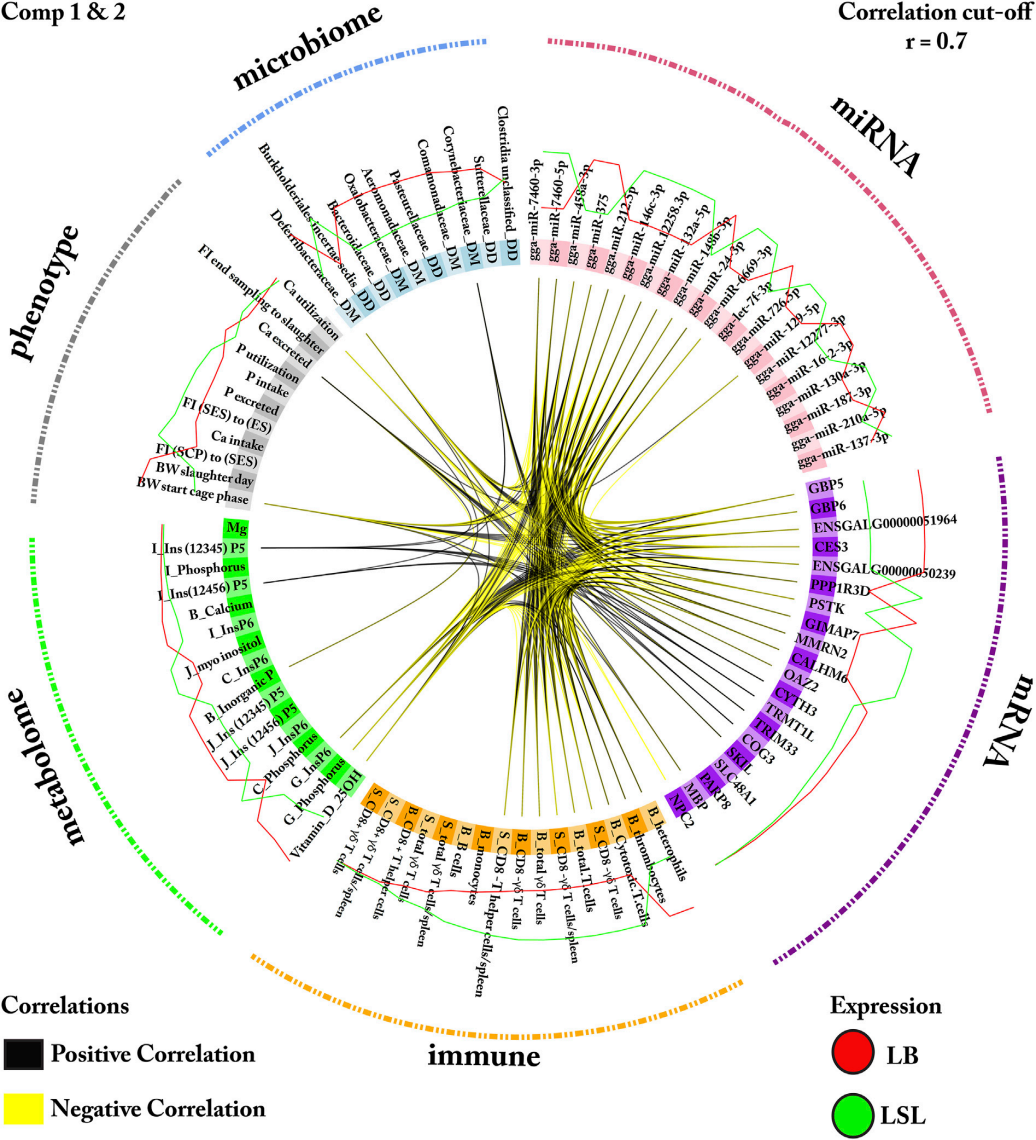
Variable plot of miRNAs, mRNAs, phenotypic, immune traits, metabolites, and microbes related to LB and LSL strains. Circos plot demonstrates the bio-signature from multiple datasets over the two components. The selected biomarkers were represented in the inner circle. Similarly, the pink, purple, orange, green, gray, and blue dashed lines outside the Circos indicate each data type. The black link suggests a positive correlation, while the yellow link depicts a negative correlation. The red and green lines represent the feature expression level in LB and LSL, respectively.

Significant features for distinguishing LB and LSL hens were selected based on their loading weights and correlation between the two components for each block. The loadings were represented as a bar plot that indicates each selected feature’s contribution on each data block: mRNA, miRNA, phenotype, immune traits, metabolome, and microbiota (Figures 3, 4). Out of these sets, for miRNAs 13/20 (65%), mRNAs 6/20 (30%), immune cells 14/16 (88%), microbes 1/10 (10%), metabolites 4/16 (25%), and phenotypic traits 6/11 (55%) were most prevalent in LSL based on the two components, as shown in Figures 3, 4. The results suggest that strain-discriminating immune features and miRNAs were prominently abundant in LSL, whereas microbes, blood/digesta parameters, and mRNAs were visibly more abundant in LB. The Circos plot demonstrates the correlation among the selected biomarker set and their profiling in LB and LSL (Figure 5).

### Bio-Signature miRNAs and Their Negatively Correlated DE Target mRNAs

**FIGURE 6:**
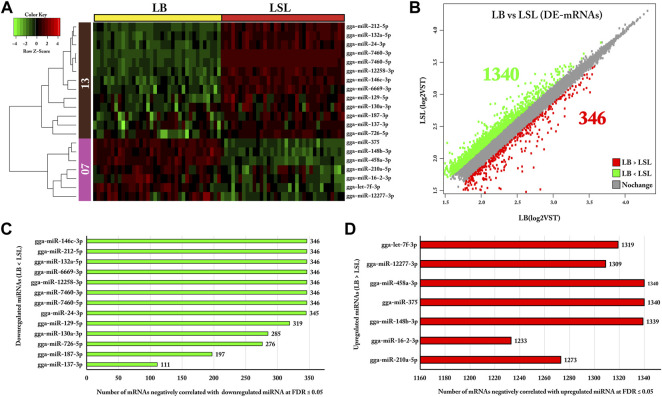
Twenty bio-signature miRNAs, their negatively correlated DE target transcripts, and differentially expressed genes in contrast for LB vs. LSL. **(A)** Heatmap representing the LB and LSL expression profiles of 20 shortlisted miRNAs from the mixOmics. According to their co-expression, these 20 miRNAs clustered apart in two groups, 13 and seven, in clusters one and two, respectively. The green color represents downregulation, while the red indicates upregulation. **(B)** Scatter plot showing differentially expressed mRNAs between LB and LSL (cutoff |VST ≥2, logFC ≥1.2|). The green and red colors indicated the downregulated and upregulated transcripts in LB, respectively, and the gray color indicated no significant difference in expression between the strains. **(C)** Bar chart showing the downregulated miRNAs in LB and upregulated in LSL and the number of negatively correlated target transcripts at FDR ≤5%. **(D)** Bar chart representing the upregulated miRNAs in LB and downregulated in LSL and the number of negatively correlated target transcripts at FDR ≤ 5%.

The co-expression clustering analysis of 20 miRNAs selected as features revealed clusters composed of 13 miRNAs and seven miRNAs in clusters 1 and 2, respectively (Figure 6A). Results show that 65% (13/20) of the miRNAs were downregulated, and the remaining 35% (7/20) were upregulated in LB (Figure 6A). Furthermore, differential expression (DE) analysis on the complete set of mRNAs revealed 1,686 transcripts were differentially expressed between LB and LSL, with about 80% of them (1,340/1,686) downregulated and about 20% (346/1,686) of them upregulated in LB compared to LSL (Figure 6B).

In addition, we performed the Pearson correlation analyses between bio-signature miRNAs and their DE putative target genes, as shown in Figure 1B. The DE mRNAs negatively correlated with bio-signature miRNAs at FDR ≤ 5% and revealed that 13 downregulated miRNAs in LB were negatively correlated with 3,956 genes, while seven upregulated miRNAs in LB were negatively correlated with 9,153 genes as shown in Figures 6C,D.

### Functional Enrichment Analysis of DE mRNAs Negatively Correlated With Bio-Signature miRNAs

**FIGURE 7:**
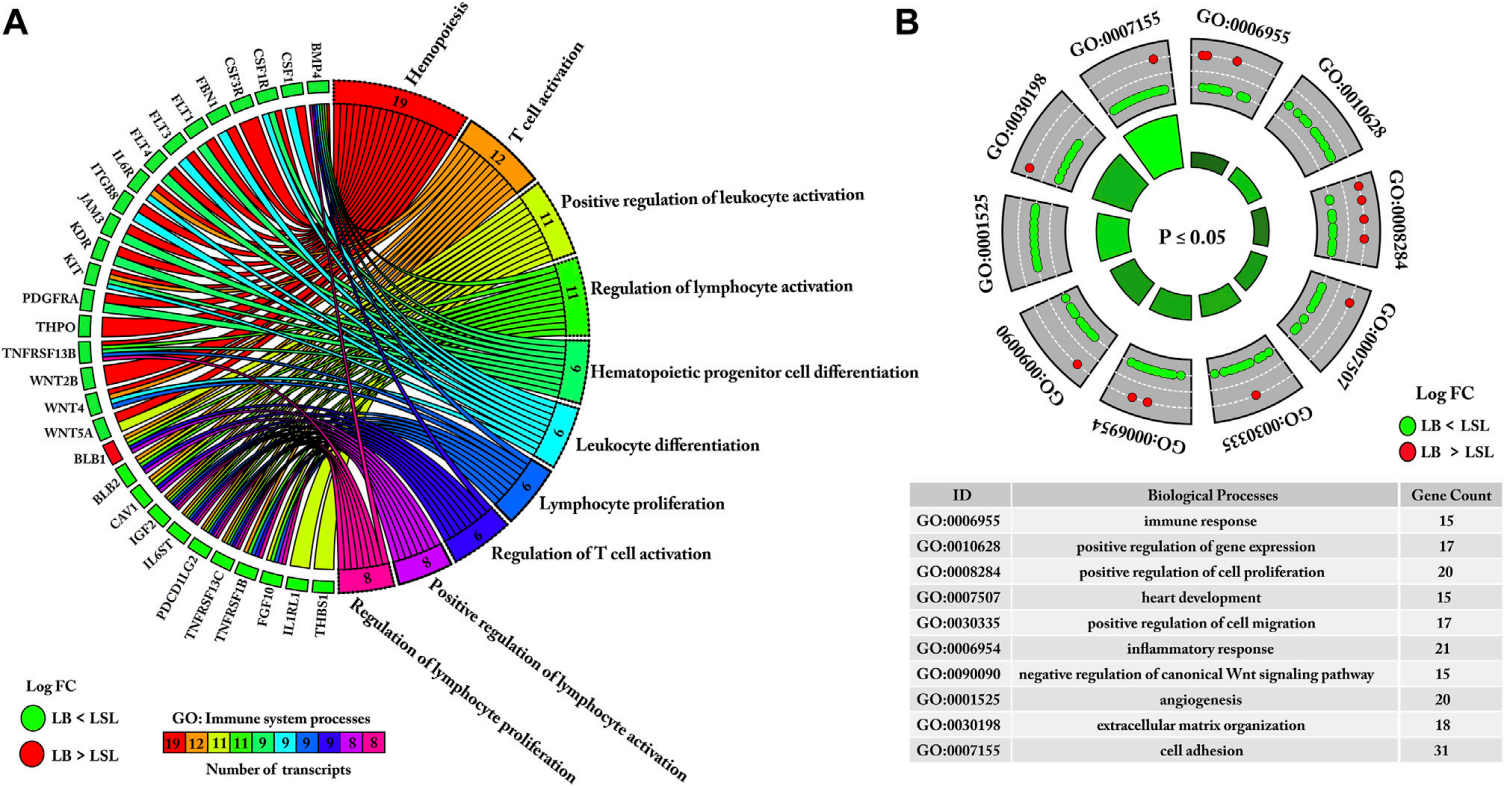
Gene ontology enrichment analysis of DE mRNAs negatively correlated with bio-signature miRNAs. **(A)** miRNAs and their negatively correlated DE target genes were subjected to enrichment analysis of gene ontology (GO) (immune system processes). The inner part of the Circos plot represents links of DE genes and identified GO terms. **(B)** Gene Ontology (biological processes) enrichment analysis was performed on bio-signature miRNAs and their negatively correlated target genes. The inner white dotted line represents the partition of logFC from lower to higher levels. The green is downregulated in LB and the red is upregulated in LB. Immune system processes and biological processes with *p* ≤ 0.05 were considered significant.

Initially, bio-signature miRNAs and their negatively correlated target genes were subjected to ClueGO and Cluepedia for gene ontology enrichment analyses (immune system processes). Immune system processes were enriched with 30 DE genes, including T-cell activation or regulation, hemopoiesis, lymphocyte activation or proliferation, and leukocyte activation or differentiation. Interestingly, 29/30 genes were downregulated in LB but upregulated in LSL; these findings indicated that genes involved in the respective immune system processes were highly expressed in LSL compared to LB (Figure 7A).

Moreover, gene ontology analysis (biological processes) was performed using DAVID. We shortlisted ten biological processes related to immune response, regulation of cell proliferation, inflammatory response, and cellular processes based on 189 DE mRNAs. The majority of these genes (∼93% 175/189) were upregulated in LSL and downregulated in LB. The remaining 14 upregulated genes in LB are also involved in the aforementioned biological processes, as shown in Figure 7B.

**FIGURE 8:**
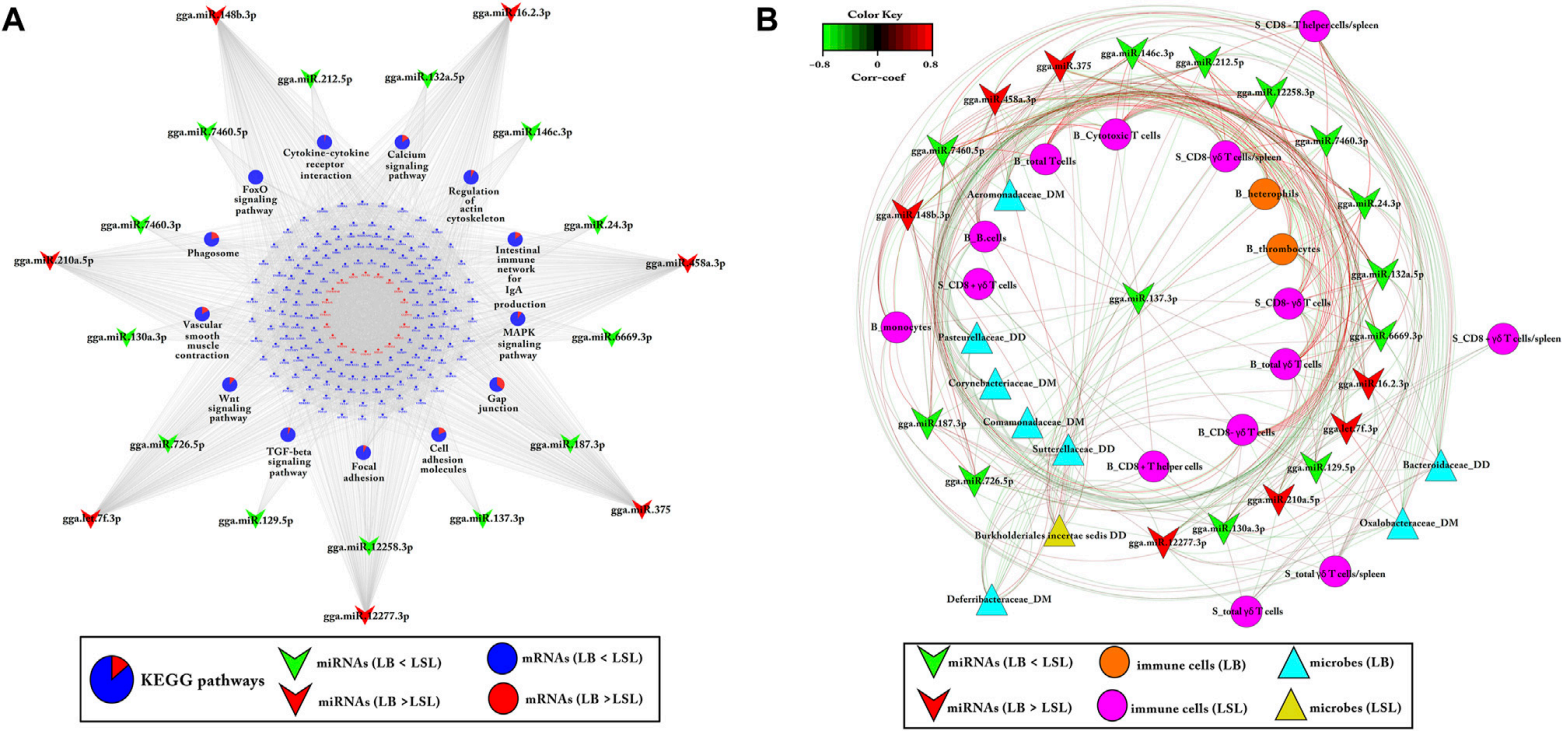
Correlation network analysis of selected features for miRNAs, immune cells, and microbes, and KEGG pathway enrichment analysis of these miRNAs and their negatively correlated DE target transcripts. **(A)** KEGG pathway enrichment analysis of bio-signature miRNAs and their negatively correlated DE target genes (*p* ≤ 0.05). The pie charts indicate the proportion of the DE gene’s contribution to KEGG pathways. The blue ellipse showed that mRNAs were downregulated in LB and upregulated in LSL, while the red ellipse showed the opposite. The green symbol depicts miRNAs downregulated in LB and upregulated in LSL, whereas the red symbol indicates those upregulated in LB and downregulated in LSL. **(B)** Correlation network derived from Pearson correlations calculated between bio-signatures of miRNAs, immune cells, and microbes (FDR ≤ 5%). Circular shape showed immune cells linked to LB (orange) or LSL (pink). Likewise, the sky blue triangle indicated microbial taxa linked with LB, while the yellow triangle showed microbial taxa associated with LSL. The red and green connections indicated the positive and negative correlation, respectively.

Moreover, KEGG pathways enrichment analysis of bio-signature miRNAs and their negatively correlated transcripts were performed (Figure 8A). In total, 13 KEGG pathways were found to be enriched (*p*-value ≤ 0.05). Most of the downregulated genes in LB and upregulated genes in LSL were enriched in immune-related pathways, including cytokine–cytokine receptor interaction, intestinal immune network for IgA production, the MAPK signaling pathway, the TGF-β signaling pathway, the FoxO signaling pathway, the Wnt signaling pathway, and others as represented in Figure 8B. In contrast, a lower proportion of transcripts, upregulated in LB and downregulated in LSL, were enriched in Gap junction, phagosome, the Ca signaling pathway, and cell adhesion molecule. Surprisingly, these findings also depict that transcripts enriched in immune-related KEGG pathways were more abundant in LSL than LB (Figure 8A).

We additionally performed correlation network analysis revealing that most immune cells were positive correlated with miRNAs in LSL including miR-7460-5p, miR146c-3p, miR-212-5p, and miR-24-3p, while microbes were more connected to LB (Figure 8B).

### Strain-Specific Bio-Signature of Immune Cells and Functional Enrichment Analysis of Correlated Gut mRNAs Transcripts

**FIGURE 9:**
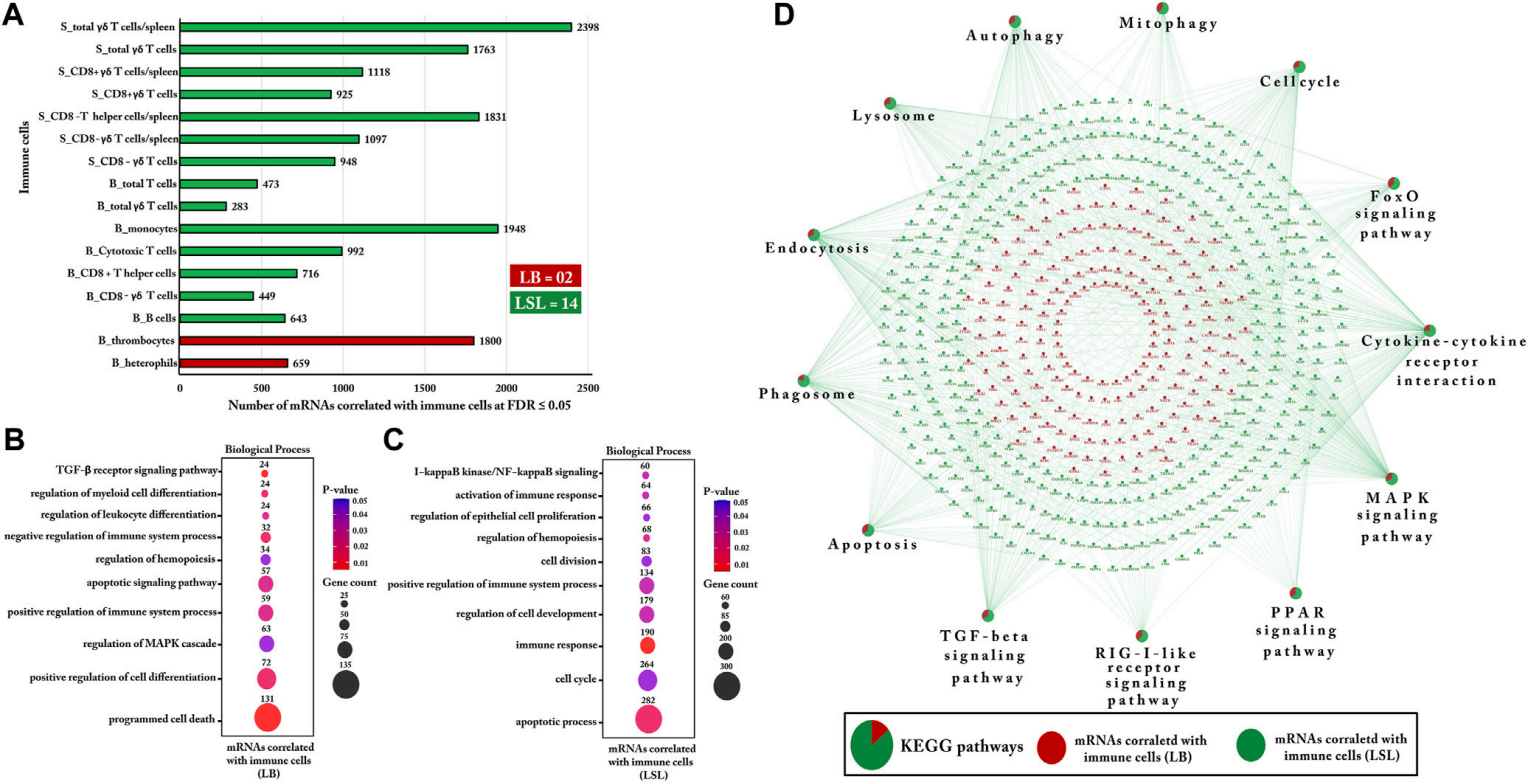
Gene Ontology and KEGG pathways enrichment analysis of mRNAs correlated with immune cells within LB and LSL. **(A)** The bar chart indicates the number of mRNAs correlated with immune cells at FDR ≤ 5% within LB and LSL (|r ≥ 0.5|). The green bar shows the immune cells that are more abundant in LSL, while the red bar depicts immune cells that are more abundant in LB. **(B)** The transcripts correlated with immune cells in LB were subject to DAVID (version 6.8) for Gene Ontology (biological processes) enrichment analysis. **(C)** Gene Ontology (biological processes) enrichment analysis of transcripts correlated with immune cells in LSL. The dot size represents the number of transcripts involved in each biological process, while the dot’s color indicates the *p*-value. **(D)** KEGG pathway enrichment analysis of mRNAs correlated with immune cells within LB and LSL. The pie charts indicate the strain-specific proportions of mRNAs correlated with immune cells to the KEGG pathways. The red ellipse shows mRNAs correlated with immune cells in LB, and the green ellipse depicts mRNAs correlated with immune cells in LSL. KEGG pathways and biological processes with *p* ≤ 0.05 were considered significant.

Sixteen immune cell types were selected as features for LB and LSL using mixOmics. About 88% of these cell types, 14/16, were shown to be more abundant in LSL, including immune cell types in blood (B cells, CD8^+^ T helper cells, cytotoxic T cells, total T cells, and monocytes), in the spleen (CD8^−^ γδ T cells/g, CD8^−^ T-helper cells/g, CD8^+^ γδ T-cells/g, total γδ T cells/g, and CD8^+^ γδ T cells), and both in blood and spleen (total γδ T cells and CD8^−^ γδ T cells). The remaining 12 percent was shown to be abundant in LB: blood heterophils and thrombocytes. Subsequently, Pearson correlation was calculated between selected immune features for LB and LSL with the complete set of mRNA profiles from the jejunum (Figure 9A).

In LB, the transcripts correlated with blood thrombocytes and heterophils were used to predict significantly enriched biological processes (gene ontology; *p*-value ≤ 0.05). Immune-related biological processes were identified, including programmed cell death, regulation MAPK cascade, cell differentiation, immune system regulation, the TGF-β receptor signaling pathway, and others shown in Figure 9B. In LSL, the transcripts correlated with 7/14 (50%) immune cells from blood and 7/14 (50%) immune cells from the spleen were also used to examine the significantly enriched biological processes. Similarly, our results indicate that most of the transcripts correlated with immune cells in LSL were predominantly involved in immune-related biological processes, including apoptotic processes, cell cycle, immune response, regulation of immune system processes, cell proliferation, activation of the immune response, and others represented in Figure 9C.

In addition, KEGG pathways of genes correlated with immune cells within LB and LSL were analyzed and revealed the enrichment in immune-related and mitochondrial dysfunctional pathways, including autophagy, mitophagy, endocytosis, phagosome, apoptosis, TGF-β signaling pathway, cytokine–cytokine receptor interaction, cell cycle, and others mentioned in Figure 9D (*p* ≤ 0.05). Interestingly, the major portion of these enriched pathways was covered by the transcripts highlighted in the LSL strain. At the same time, the transcripts correlated with immune cells in LB were also involved in these pathways, but to a lesser extent than in LSL.

### Strain-Specific Bio-Signature of Blood/Digesta Parameters and Functional Enrichment Analysis of Correlated Gut mRNAs Transcripts

**FIGURE 10:**
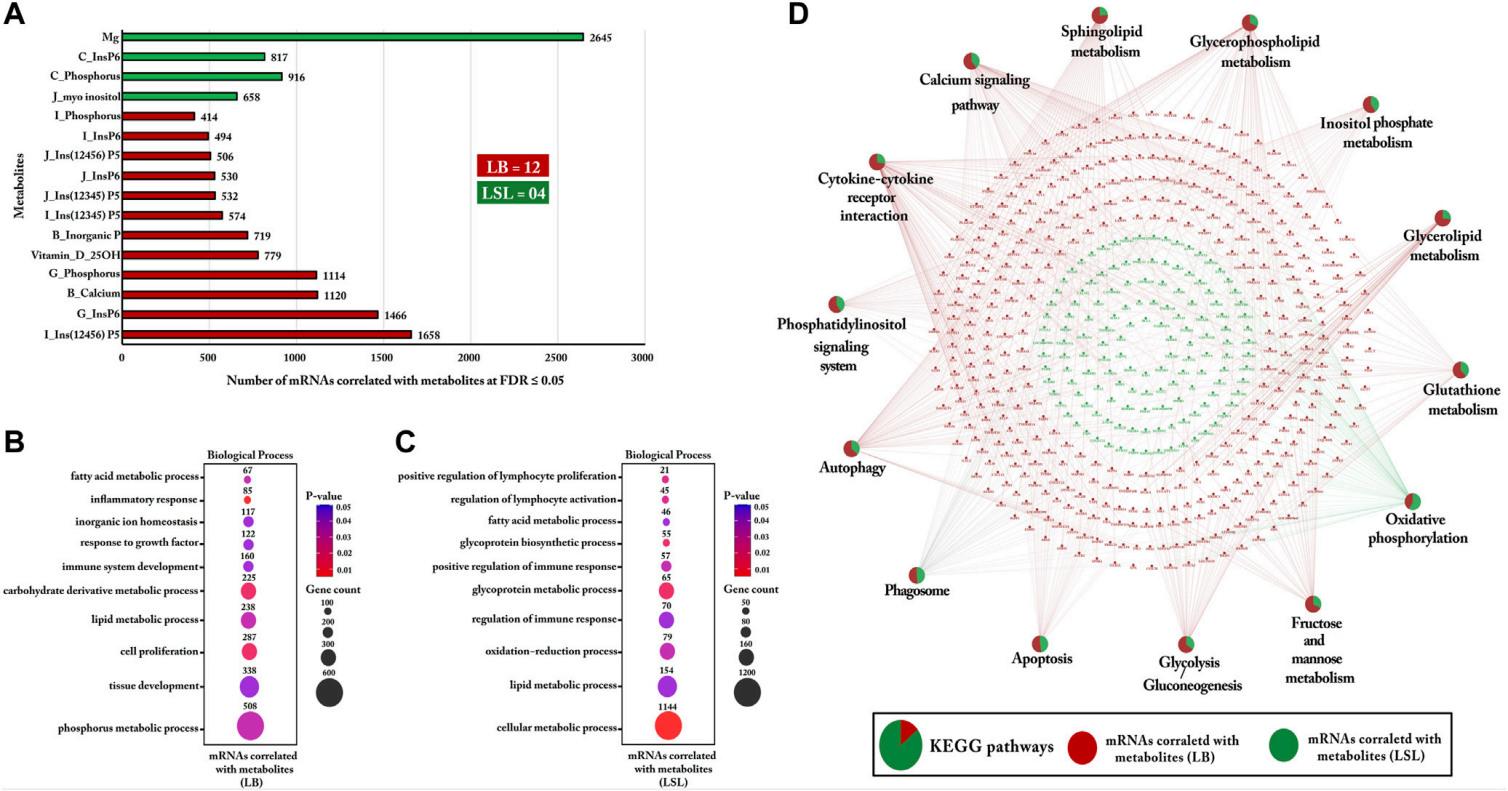
Gene Ontology and KEGG pathways enrichment analysis of mRNAs correlated with blood/digesta parameters within LB and LSL. **(A)** The bar chart indicates the number of mRNAs correlated with blood/digesta parameters at FDR ≤ 5% within LB and LSL (r ≥ 0.5). The green bar shows the blood/digesta parameters that are more abundant in LSL, while the red bar depicts blood/digesta parameters that are more abundant in LB. **(B)** The transcripts correlated with blood/digesta parameters in LB were subjected to DAVID (version 6.8) for Gene Ontology (biological processes) enrichment analysis. **(C)** Gene Ontology (biological processes) enrichment analysis of transcripts correlated with blood/digesta parameters in LSL. The dot size represents the number of transcripts involved in each biological process, while the dot’s color indicates the *p*-value. **(D)** KEGG pathway enrichment analysis of mRNAs correlated with blood/digesta parameters within LB and LSL. The pie charts indicate the strain-specific proportions of mRNAs correlated with blood/digesta parameters to the KEGG pathways. The red ellipse shows mRNAs correlated with blood/digesta parameters in LB, and the green ellipse depicts mRNAs correlated with blood/digesta parameters in LSL. KEGG pathways and biological processes with *p* ≤ 0.05 were considered significant.

Sixteen blood/digesta parameters (metabolites, P, Ca, MI, and hormones) were shortlisted as features for LB and LSL. About 76% (12/16) of these blood/digesta parameters were more abundant in LB: MI (jejunum), Ins (1,2,3,4,5) P_5_ and Ins (1,2,4,5,6) P_5_ (jejunum and ileum), InsP_6_ (jejunum and gizzard), P (ileum and gizzard), and Ca, inorganic P, and vitamin-D-25OH (plasma). The remaining 14% (4/16) was shown to be more abundant in LSL: Mg (Plasma), MI (jejunum), P, and InsP_6_ (crop). Subsequently, Pearson correlation was calculated between selected blood/digesta parameters for LB and LSL with the complete set of mRNA profiles from the jejunum (Figure 10A).

Based on the correlated transcripts, gene ontology (biological processes) enrichment analyses were performed. In LB, these genes were primarily enriched in metabolism, development, and immune systems, such as the P, lipid, and carbohydrate derivative metabolic processes, immune system development, inflammatory response, tissue development, growth factors, cell proliferation, and inorganic ion homeostasis (Figure 10B). The most enriched biological process was the P metabolic process, which included 508 correlated transcripts (*p*-value = 0.03).

In LSL, the results revealed that the correlated transcripts were mainly involved in metabolism and immune functions, such as cellular metabolic process, lipid metabolic process, glycoprotein metabolic process, fatty acid metabolic process, lymphocyte activation, proliferation processes, and regulation of immune response (Figure 10C). The predominantly enriched biological process was the cellular metabolic process containing 1,144 correlated mRNAs (*p*-value = 0.01).

Similarly, KEGG pathway analyses were performed with the transcripts correlated with blood/digesta parameters. The transcripts were enriched in metabolic and immune pathways, including glycerophospholipid metabolism, inositol phosphate metabolism, glycerolipid metabolism, glutathione metabolism, fructose, and mannose metabolism, autophagy, phagosome, apoptosis, and cytokine–cytokine receptor interaction (Figure 10D). In addition, we found that transcripts derived from parameters more abundant in the LB strain dominated the enrichment of pathways compared to LSL, including sphingolipid metabolism, glycerophospholipid metabolism, cytokine–cytokine receptor interaction, glycolysis, and gluconeogenesis (Figure 10D).

### Strain-Specific Bio-Signature of Microbes and Functional Enrichment Analysis of Correlated Gut mRNAs Transcripts

**FIGURE 11:**
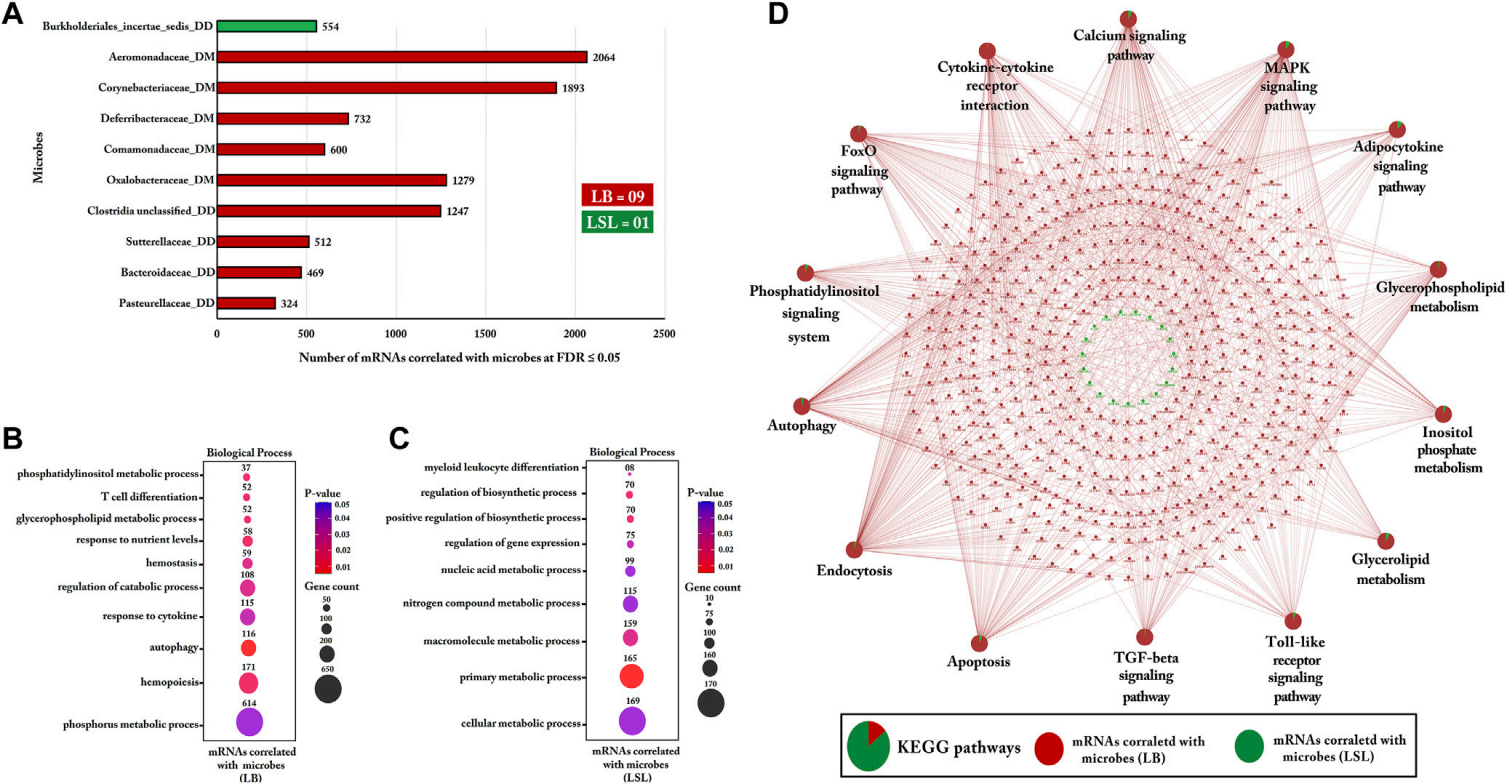
Gene Ontology and KEGG pathways enrichment analysis of mRNAs correlated with duodenal microbiota within LB and LSL. **(A)** The bar chart indicates the number of mRNAs correlated with microbes at FDR ≤ 5% within LB and LSL (r ≥ 0.5). The green bar shows more abundant microbes in LSL, while the red bar depicts more abundant microbes in LB. **(B)** The transcripts correlated with microbes in LB were subject to DAVID (version 6.8) for Gene Ontology (biological processes) enrichment analysis. **(C)** Gene Ontology (biological processes) enrichment analysis of transcripts correlated with microbes in LSL. The dot size represents the number of transcripts involved in each biological process, while the dot’s color indicates the *p*-value. **(D)** KEGG pathway enrichment analysis of mRNAs correlated with microbes within LB and LSL. The pie charts indicate the strain-specific proportions of mRNAs correlated with microbiota to the KEGG pathways. The red ellipse shows mRNAs correlated with microbes in LB, and the green ellipse depicts mRNAs correlated with microbes in LSL. KEGG pathways and biological processes with *p* ≤ 0.05 were considered significant.

Ten microbes were shortlisted as features for LB and LSL from duodenal digesta/mucosa. About 90% (9/10) of these microbial taxa were more abundant in LB: Aeromonadaceae, Bacteroidaceae, Clostridia, Comamonadaceae*,* Corynebacteriaceae, Deferribacteraceae*,* Oxalobacteraceae, Pasteurellaceae, and Sutterellaceae. The remaining 10% (1/10) was abundant in LSL: Burkholderiales. Subsequently, Pearson correlation was calculated between selected microbes for LB and LSL with the complete set of mRNA profiles from the jejunum (Figure 11A).

For gene ontology analysis (biological processes), the transcripts identified for LB revealed enrichment in metabolic and immune functions such as the phosphatidylinositol metabolic process, glycerophospholipid metabolic process, P metabolic process, hemostasis, T- cell differentiation, and cytokine response (Figure 11B). The most enriched biological process was the P metabolic process comprising 614 correlated transcripts (*p*-value = 0.04).

In LSL, the 554 transcripts correlated with Burkholderiales from duodenal digesta were used to investigate the function of these transcripts. The role of these 554 genes was observed in metabolic processes, including the cellular metabolic process, primary metabolic process, macromolecule metabolic process, nitrogen compound metabolic process, and nucleic acid metabolic process (Figure 11C).

KEGG pathway analysis was performed for the same correlated gene sets of LB and LSL. The transcripts correlated with microbes within LB were predominantly involved in immune or metabolic signaling pathways, covering Ca, MAPK, adipocytokine, Toll-like receptor, TGF-β, FoxO, and phosphatidylinositol as well as metabolic pathways, including glycerophospholipid, inositol phosphate, and glycerolipid metabolism (Figure 11D). In contrast, the proportion of transcripts correlated with microbes within LSL was too low for consideration.

### Strain-Specific Bio-Signature of Phenotypic Traits and Functional Enrichment Analysis of Correlated Gut mRNAs Transcripts

**FIGURE 12:**
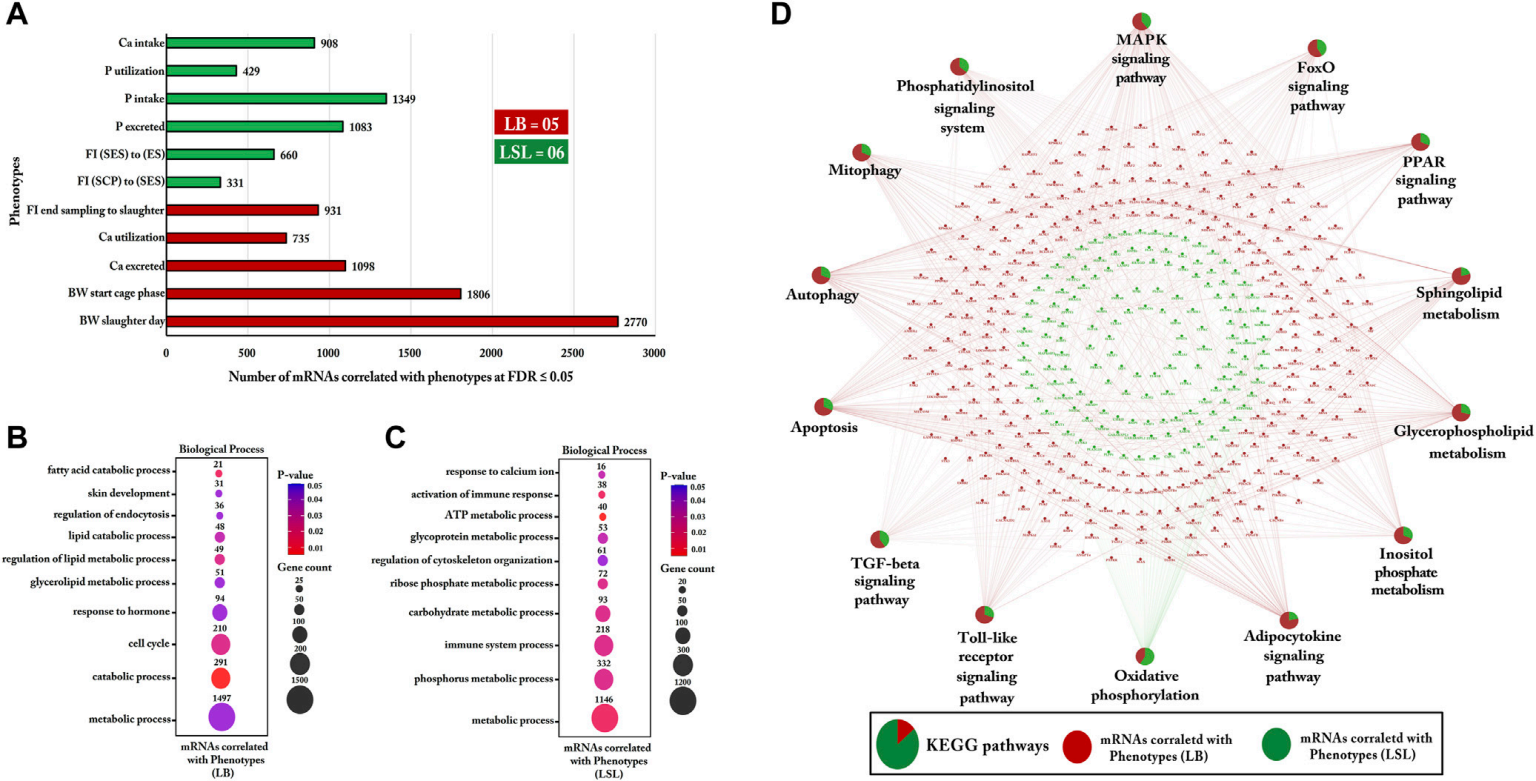
Gene Ontology and KEGG pathways enrichment analysis of mRNAs correlated with phenotypic traits within LB and LSL. **(A)** The bar chart indicates the number of mRNAs correlated with phenotypic traits at FDR ≤5% within LB and LSL (r ≥ 0.5). The green bar shows more abundant phenotypic traits in LSL, while the red bar depicts more abundant phenotypic traits in LB. **(B)** The transcripts correlated with phenotypic traits in LB were subjected to DAVID (version 6.8) for Gene Ontology (biological processes) enrichment analysis. **(C)** Gene Ontology (biological processes) enrichment analysis of transcripts correlated with phenotypic traits in LSL. The dot size represents the number of transcripts involved in each biological process, while the dot’s color indicates the *p*-value. **(D)** KEGG pathway enrichment analysis of mRNAs correlated with phenotypic traits within LB and LSL. The pie charts indicate the strain-specific proportions of mRNAs correlated with phenotypic traits to the KEGG pathways. The red ellipse shows mRNAs correlated with phenotypic traits in LB, and the green ellipse depicts mRNAs correlated with phenotypic traits in LSL. KEGG pathways and biological processes with *p* ≤ 0.05 were considered significant.

Interestingly, all eleven phenotypic traits used as input were selected as features that differ between LB and LSL. The traits Ca intake, P utilization, P intake, P excretion, feed intake (start excreta sampling to end sampling), and feed intake (start cage phase to start excreta sampling) had higher values in LSL. The remaining 45% was higher in LB than in LSL: Feed intake (31 weeks), Ca utilization, Ca excretion, bodyweight (start cage phase), and bodyweight (31 weeks). Subsequently, Pearson correlation was calculated between selected phenotypic traits for LB and LSL with the complete set of mRNA profiles from the jejunum (Figure 12A).

In LB, the transcripts correlating with the bio-signature of phenotypic traits were used to predict the functions and pathways. The correlated genes are involved in metabolic and catabolic biological processes, including the glycerolipid metabolism, lipid metabolism, lipid catabolic process, and fatty acid catabolic process (Figure 12B). The most noticeable enriched biological process was a metabolic process comprising 1,497 correlated transcripts (*p*-value = 0.03).

In LSL, most of the correlated genes were involved in metabolism and the immune system, such as P metabolism, carbohydrate metabolism, glycoprotein metabolism, ATP metabolic process, ribose phosphate metabolism, and immune responses (Figure 12C). One of the most enriched biological processes was the P metabolic process, including 1,146 correlated transcripts (*p*-value = 0.02).

For KEGG pathway analysis, the proportion of correlated transcripts within LB was more enriched than LSL for mitophagy, autophagy sphingolipid metabolism, the PPAR signaling pathway, apoptosis, the Toll-like receptor signaling pathway, and the adipocytokine signaling pathway (Figure 12D).

## Discussion

Over the past decade, data integration methods have become increasingly popular due to the plethora of biological data generated from different biological experiments (Gligorijević and Pržulj, 2015). A multi-omics data integration approach can identify novel biomarkers and gain profound insight into biological mechanisms when integrating data from different experimental designs (Graw et al., 2021). In the present study, we applied a muti-omics data integration approach to analyze the miscellaneous dataset; our results established that the two strains were noticeably different regarding their immune system, transcriptional responses, metabolism, gut microbial activity, and physiological traits such as development and body weight, although both layer lines had comparable egg production performance. Moreover, mix-omics provided a shortlist of significant bio-signatures from pan-omics data comprising 20 miRNAs, 20 mRNAs, 16 immune cell types, 10 intestinal microbes, 11 phenotypic traits, and 16 blood/digesta parameters (metabolites, P, Ca, MI, and hormones). These biomarkers of the twolayer strains revealed distinct modes of adaptation in metabolic and immune pathways.

The mRNAs transcripts are the master regulator in almost every biological process and pathway (Mattick et al., 2010). In the current study, 20 mRNAs were categorized as bio-signature different between LB and LSL. 14/20 were upregulated in LB and downregulated in LSL, while 6/20 were downregulated in LB and upregulated in LSL (Figure 3 and Figure 4). Guanylate-binding proteins (GBPs) are the major component of cellular immunity and are pivotal in controlling intercellular infections (Sohrabi et al., 2018). Previously, a study indicated that copy number variations of *GBP2* and *GBP4* are related to growth traits in Chinese domestic cattle (Hao et al., 2020). An earlier study revealed that viremia levels and weight gain in response to PRRSV infection in pigs are affected by genotype-dependent alterations in *GBP5* and *GBP6* expression (Kommadath et al., 2017). Another study indicated that *GBP5* is involved in host defense, the assembly of inflammasomes, and inflammatory responses against pathogenic bacteria in *GBP5* knockout mice (Shenoy et al., 2012). Interestingly, our study also found both of these genes as highly expressed biomarkers in the LB strain. The myelin basic protein (*MBP*) and its related transcripts are widely expressed in cells of the immune system, such as T lymphocytes, B lymphocytes, and macrophages (Feng, 2007; Xu et al., 2016). Previously, a study indicated that the bone marrow and the immune system contain MBP-related transcripts and are predominantly expressed in T cells (Marty et al., 2002; Xu et al., 2016). In the present study, we identified the *MBP* gene as a highly expressed biomarker in the LSL strain.

There is considerable evidence that miRNAs are highly conserved among species and significantly regulate gene expression (Zhang et al., 2012; O'Brien et al., 2018). In the present study, we focus on 20 miRNA biomarkers and their mRNA targets that differ between LB and LSL strains. Regarding biological processes related to the immune system, transcripts inversely correlating with bio-signature miRNAs were predominantly higher in terms of expression in LSL than in LB, which was valid for 29 out of the 30 identified transcripts. The related processes include hemopoiesis, T-cell activation, lymphocyte activation, proliferation, leukocyte differentiation, and activation. The overrepresentation of transcripts assigned to immune system processes in LSL compared to LB provides additional evidence to previous studies (Habig et al., 2012, 2014; Iqbal et al., 2021; Ponsuksili et al., 2021). Stressors affected the heterophil-to-lymphocyte (H/L) ratio, which can be used to assess the level of stress imposed on laying hens (Gross and Siegel, 1983). An earlier study indicated that according to H/L ratio calculations, LB hens had ratios 2.6-fold higher than the LSL hens, and the H/Lratios of LB hens indicate prolonged stress exposure. In addition, previous studies argue that LSL hens seem to have a more adaptive immunological phenotype, while LB hens have a distinct innate immunological phenotype (Hofmann et al., 2021), and it was also shown in this study that T-cell activation, lymphocyte activation, and proliferation are higher in LSL hens. Stress influences the immune system to downregulate its responsiveness (Habig et al., 2014; Monson et al., 2018; Abbas et al., 2020; Goel et al., 2021). Accordingly, it is conceivable that higher susceptibility of LB hens to stress might be responsible for the lower abundance of transcripts related to immune function compared to LSL.

MiR-375 has been reported to be highly enriched in intestinal endocrine cells (EECs), and these cells play an essential role in systemic energy homeostasis (Hung and Sethupathy, 2018). A higher level of miR-375 was also found in the jejunal mucosa of the LB hens, which is consistent with our previous findings that it may be associated with the higher growth rate of LB compared to LSL (Ponsuksili et al., 2021). Our findings show that miR-375 is less abundant in LSL than in LB and can upregulate 29 transcripts significantly associated with immunity, as shown in Figures 7A, 8A. The abundance of miR-375 in the gut is also strongly negatively correlated with total T cells, total γδ T cells, and CD8^−^ γδ T cells (r < −0.7; FDR < 10^−11^) and positively correlated with thrombocytes in the blood. Recently, a study in mice reported that miR-375 might improve immune functions by regulating Kupffer cells (Ke et al., 2019). We speculate that miR-375 is likely to be a new therapeutic target for immune-mediated diseases in layer chickens. Another study revealed that miR-148b-3p was downregulated in LSL and upregulated in LB. This miRNA contributes to osteogenic differentiation and bone remodeling (Manochantr et al., 2017). For the let-7f miRNA, a recent study suggests that let-7f functions as a crucial component of the miRNA network regulating immunity (Kumar et al., 2015). Consistent with these findings, our results showed that let-7f-3p is downregulated in LSL, and its potential targets are concomitantly upregulated and govern the immune cell activation, proliferation, and differentiation processes in the gut. Furthermore, we identified bona fide gene noggin (*NOG*) that was upregulated in LB and downregulated in LSL; this gene plays an essential role in body tissue development such as muscle and bones. *NOG* regulates the TGF-β signaling pathway, which plays a significant role in bone development by stimulating osteoprogenitor enrichment (Wu et al., 2016).

Signal transduction in complex immune responses is triggered by *TNFRSF13B/TNFRSF13C* combined with *TNFSF13B* (Maeda et al., 2014). The expression of TNF Receptor Superfamily Member 13 B/C (*TNFRSF13B and TNFRSF13C*) was higher in LSL and may play an important role in humoral immunity by regulating the intestinal immune system network for the IgA production signaling pathway (Figure 8B).

B-cell survival and maturation are dependent on this *TNFSF13B/TNFRSF13C* system. Earlier studies showed that blocking *TNFSF13B/TNFRSF13C* signaling may effectively treat autoimmune diseases mediated by B-cells in humans (Ferrer et al., 2014). As a result, we speculate that *TNFSF13B/TNFRSF13C* are two essential components of gut immunity among laying hens. Macrophages play a crucial role in innate and acquired immune systems and are an integral component of the mononuclear phagocytic system (Elhelu, 1983). Macrophages need cytokines for their proper functioning (Arango Duque and Descoteaux, 2014). We identified two upregulated genes in LSL, colony-stimulating Factor 1 and receptor for colony-stimulating factor 1 (*CSF1* and *CSF1R*), which were downregulated in LB. The protein encoded by the *CSF1* gene is a cytokine. The *CSF1/CSF1R* system plays a pivotal role in controlling macrophages’ development, differentiation, and function and regulates the cytokine–cytokine receptor interaction pathway. Therefore, the higher abundance of *CSF1/CSF1R* in LSL might contribute to the more pronounced acquired immunity compared with the LB strain. We also identified interleukin-cytokine receptor (*IL1R2, IL1RAP*, and *IL1RL1*) upregulation in LSL. Earlier studies have indicated that the immune response and inflammation are triggered by IL-1, secreted by macrophages, fibroblasts, B cells, granular lymphocytes, endothelium, and astrocytes (Carmi et al., 2009; Sims and Smith, 2010). Overall, significant differences were found in the miRNA- and mRNA-transcript profiles of the jejunum of the two strains. In particular, we shortlisted some essential biomarkers, which are the regulators of immune-related and developmental pathways. Given the high genetic differentiation of the two strains (Heumann-Kiesler et al., 2021), the data provide important insights into the interplay between miRNA and mRNA, which represent different strategies adopted at the molecular level to achieve optimal performance.

Previously, the genetic selection of poultry aimed to improve feed conversion and/or egg-laying performance. In addition, chickens vary in weight gain along a productive period. Recently, data have shown that the immune system varies during the hens’ production period due to genetic differences (Koenen et al., 2002; Kjærup et al., 2017; Hofmann et al., 2021). One of the data sets included in the present study comprised T-lymphocytes, B-lymphocytes, heterophils, monocytes, and thrombocytes from blood, the spleen, and cecal tonsils of LB and LSL strains. Our feature selection results indicated that most of the T-lymphocytes and B-lymphocytes from the blood and spleen were more abundant in LSL, while only thrombocytes and heterophils from the blood were higher in LB than in LSL. Higher proportions of thrombocytes and heterophils have been previously detected in the blood of LB than LSL (Schmucker et al., 2021). Also, there is evidence that chicken thrombocytes may have an immunological function similar to that of mammalian platelets (St. Paul et al., 2012; Ferdous and Scott, 2015).

Compared to other cells, thrombocytes are the primary carrier of TGF-β in the body and contain 40 to 100 times more TGF-β (Karolczak and Watala, 2021), and platelet-thrombocyte number and the TGF-β concentration are positively correlated in peripheral blood (Weibrich et al., 2002; Lu et al., 2017; Guo et al., 2019). Interestingly, our results suggested that genes correlated with thrombocytes and heterophils in the LB strain were primarily involved in immune-related pathways, including TGF-β receptor signaling pathways, the apoptotic signaling pathway, regulation of MAPK cascade, and regulation of the immune system process (Figure 9B). We also detected a positive correlation between thrombocytes in blood with the *TGFB1*, *TGFB2*, *TGFB3*, and *TGFBR1* genes with essential roles in regulating the TGF-β signaling pathway. These findings suggest that thrombocytes may be active carriers of the TGF-β molecules. However, thrombocytes contribute to various functions within the immune system, cell differentiation, apoptosis, and cellular homeostasis, but the contribution of thrombocytes is not well documented in birds yet, thus highlighting the relevance of studying the connections between thrombocytes and TGF-β as a molecular mechanism in bird immunology. In avian species, three major subsets of lymphocytes compose the adaptive immune system: T and B lymphocytes and natural killer cells. Birds have a significantly higher number of circulating γδ T cells than humans and rodents. We detected a higher number of γδ T cells in the blood and spleen of the LSL strain than the LB strain (Figure 9A). We additionally identified that the genes correlated with γδ T cells significantly contributed to immune-related pathways in LSL strain, including cytokine–cytokine receptor interaction, the MAPK signaling pathway, PPAR signaling, apoptosis, autophagy, and mitophagy.

The gastrointestinal tract (GIT) harbors a complex and diverse microbiota in chicken, which plays a significant role in host health, metabolism, and immunity (Kers et al., 2018; Shang et al., 2018). Previously, studies demonstrated that gut microbiota profoundly affected chicken immune system development (Broom and Kogut, 2018; Diaz Carrasco et al., 2019). In accordance with these findings, our data suggest that genes correlated with the microbiota of mucosa and digesta were shown to be more abundant in LB than in LSL strain and were involved in the immune-related and metabolic pathways. Recently, significant correlations between cytokine gene expression (*IL-10*, *IL4*, and *IFN-γ*) and microbiota communities were observed at the early stages of chicken growth (Diaz Carrasco et al., 2019). In the intestinal tract, commensal microbes modulate cytokine production, essential for host innate and adaptive immune responses (Corthay, 2006). For instance, Clostridia was identified as a critical factor in regulating immune function (Schirmer et al., 2016). Similarly, our data from digesta highlighted that Clostridia was positively correlated with *IL20RA*, *IL22*, *IL2RB*, and *IL4R* expression in the LB strain. The *IL22* encoded protein is involved in host antimicrobial defense at the mucosal surface and is beneficial to the host intestinal inflammatory responses during infectious diseases, as shown in *IL22−/−* mice, which displayed reduced microbial diversity and slightly amplified vulnerability to host infectious diseases (Keir et al., 2020). We postulate that dynamic crosstalk between *IL22* and Clostridia may be relevant for achieving and maintaining the gut microbiota and host immunity balance. Studies have shown that different gut microbiota species are involved in the host’s defense against harmful microorganisms, such as Firmicutes, Bacteroidetes, Actinobacteria, and Proteobacteria (Gevers et al., 2012; Lloyd-Price et al., 2016).

We also identified Corynebacteriaceae (an Actinobacteria phylum member) and linked host genes enriched in autophagy and endocytosis. In addition, the autophagy-related 5 (*ATG5*) gene was positively correlated with Corynebacteriaceae and played a critical role in regulating innate and adaptive immunity. Furthermore, we identified five bacteria, Aeromonadaceae, Oxalobacteraceae, Comamonadaceae, Sutterellaceae, and Pasteurellaceae, which belong to the phylum Proteobacteria. Studies have shown that γ-proteobacteria are typical hallmarks of acute mucosal infections because of their pathogenic properties (Molloy et al., 2013; Raetz et al., 2013). Consistent with these findings, our results showed that Aeromonadaceae (a γ-proteobacteria) and genes negatively correlated with the abundance of this bacteria were involved in the Toll-like receptor signaling and apoptosis pathways. For instance, the *STAT1* transcription factor controls the responses to acute microbial infections through canonical interferon (IFN) signaling (Marié et al., 2021). We suggest a scenario where the expression of critical immune switches, for example, *STAT1*, *IFNAR1*, and *IFNAR2*, is downregulated by the pathogenic mechanisms of Aeromonadaceae and may lead to differential immune responses on the mucosal surface.

Furthermore, our results indicate that the abundance of other microbes such as Oxalobacteraceae, Comamonadaceae, Sutterellaceae*,* and Pasteurellaceae were correlated with immune pathways and metabolic pathways, including Ca signaling, adipocytokine signaling, Toll-like receptor signaling, FoxO signaling, glycerolipid metabolism, inositol phosphate metabolism, apoptosis, and TGF-β signaling pathway. However, these signaling and metabolic pathways are well known for their role in the immune system and metabolism. Compared to the results of the host gene expression profiles, where the immune system processes were predominantly highlighted in LSL, the duodenal microbiota component was predominantly on the side of the LB strain. This might underline the use of different strategies of these two strains but certainly highlights the importance of the gut microbiota for host immunity and metabolic activity.

Recently, a study indicated that magnesium (Mg) plays a pivotal role in energy production metabolic processes such as glycolysis, gluconeogenesis, and oxidative phosphorylation (Pilchova et al., 2017). In line with these findings, our results revealed that genes correlated with Mg in LSL were enriched in oxidative phosphorylation, glycolysis, and gluconeogenesis pathways. For phytate metabolism, the degradation process of InsP_6_ produces MI and lower inositol phosphates, which are involved in various immune cell functions, including proliferation, cytokine production, and cytotoxicity (Hofmann et al., 2021). Similarly, our study revealed that genes correlated with MI and InsP_6_ in LSL were enriched in lymphocyte proliferation and activation. Several studies reported that numerous transcellular and paracellular mechanisms absorb nutrients from the GIT, including the vitamin D system as a critical player in maintaining Ca and P homeostasis in the body (Delezie et al., 2015; Sommerfeld et al., 2020; Hofmann et al., 2021; Iqbal et al., 2021; Reyer et al., 2021). Correspondingly, our results stipulate that transcripts correlated with vitamin-D-25OH were enriched in P metabolic processes, inositol phosphate metabolism, and Ca signaling pathways in the LB strain. Recently, a study reported that P homeostasis is controlled by the *ITPK1* gene in Arabidopsis (Whitfield et al., 2020). Likewise, we identified Inositol Tetrakisphosphate 1-Kinase (*ITPK1*), which was positively correlated with vitamin-D-25OH and involved in the phosphate metabolism, revealing the role of *ITPK1* in phosphate homeostasis. Interestingly, we found that genes linked to InsP_6_, Ca, and P in the LB strain were involved in Ca signaling, phosphatidylinositol signaling, autophagy, apoptosis, cytokine–cytokine receptor interaction, sphingolipid metabolism, glycerophospholipid metabolism, inositol metabolism, and glycerolipid metabolism. These pathways are crucial in terms of immunity and metabolism and enlighten the importance of minerals, MI, and vitamin D in the chicken immune system and metabolic activity.

## Conclusion

Together, we provided a bio-signature feature list containing 20 miRNAs, 20 mRNAs, 16 immune parameters, 10 microbes, 11 phenotypic traits, and 16 digesta/blood parameters, which discriminate between LB and LSL. This clearly shows that in addition to a zootechnical characterization, several molecular phenotypes can be inferred, providing unique strain-specific biosignatures that reliably distinguish these two contrasting high-yielding laying hen strains. Many of these strain-specific identified features were associated with many molecular pathways. We found the gut microbiota–specific LB strain to be associated with most immune-related pathways, whereas the host miRNA– and immune cell–specific LSL strain was enriched in immune-related pathways. Integration of extensive biological datasets, including deep sequencing mRNA and miRNA expression data in jejunum mucosa, immune cells, metabolites and hormones in the blood, the microbiota in both duodenum digesta and mucosa, and physiological data, revealed host–microbiota interactions and changes in immune and metabolic systems. Our results suggest that both strains implement different intrinsic approaches, shaping their immunity and metabolic activity.

To the best of our knowledge, this is the first study to simultaneously compare the two strains of laying hens, LB and LSL, in terms of their immune system, gene expression, and microbial activity in the gastrointestinal tract, metabolism, and phenotypic traits such as body weight, mineral utilization, and response to external stimuli. Even though we used a large number of laying hens per strain (LB, *n* = 36; LSL, *n* = 35), batch effects cannot be excluded here, as the animals originate from one batch each. Our results provide the basic information that contributes to the understanding of the mechanisms underlying the immune system, metabolism, and host–microbiome interaction in laying hens.

## Data Availability

The datasets presented in this study can be found in online repositories. The names of the repository/repositories and accession number(s) can be found below: https://www.ebi.ac.uk/metagenomics/, E-MTAB-9136 and E-MTAB-9109.
